# Heritability and Variability of Quality Parameters of Tomatoes in Outdoor Production

**DOI:** 10.34133/2020/6707529

**Published:** 2020-01-28

**Authors:** Christian Zörb, Hans-Peter Piepho, Sabine Zikeli, Bernd Horneburg

**Affiliations:** ^1^University of Hohenheim, Institute of Crop Science, 70593 Stuttgart, Germany; ^2^University of Kassel, Section of Organic Plant Breeding and Agrobiodiversity, 27213 Witzenhausen, Germany

## Abstract

The tomato is the most important vegetable globally. In England, outdoor tomatoes are widely grown by amateur gardeners, with the number of their allotment plots exceeding 150000. For instance, in Germany, only about 16% of tomato plants are cultivated according to organic standards, although these rates are expected to increase. Breeding for yield and fruit quality can increase resource efficiency. Therefore, we need to evaluate the variability of yield and fruit quality parameters, to calculate the heritability of these traits and to identify superior genotypes for organic outdoor tomato production and breeding. With these aims, we grew 24 tomato genotypes of diverse origins in a two-year field trial. The heritability of quality traits such as glucose, fructose, organic acid, and lycopene was high. The medium heritability for yield suggests that trials with a larger number of locations are needed for the reliable selection of this character. Negative correlations of sugar concentrations with fruit weight and of organic acid concentration with fruit weight and yield suggest trade-offs in breeding for larger fruits and higher yields. Breeding for increased lycopene content is not subject to these challenges; the concentrations of the primary metabolite sugars and organic acids are positively correlated.

## 1. Introduction

The tomato (*Lycopersicon esculentum* Mill.) is the most important vegetable globally and in Europe [1]. Some examples of figures for Germany spotlight the situation for tomatoes in Central Europe. The tomato is the vegetable with the highest per capita consumption at 24.9 kg per year (Federal Institute for Agriculture and Food, 2015). Commercial tomato production covered 375 hectares in 2017 [2]. With respect to Central and Northern Europe, most tomatoes are imported, with the import rate being 92.8% for Germany in 2016 [1]. However, only about 16% of the domestic German production was cultivated according to organic standards [2]. This rate is expected to increase, not only in Europe, because of the rise of consumer preferences favouring organic products with regard to health and environmental benefits [3]. One of the main reasons for the current growth of organic food production is consumer demand for local food produced by safe methods with minimal negative environmental impact [4].

Tomato cropping systems range from hydroponic greenhouse systems with complete ambient control to organic outdoor production. Greenhouse gas emissions (in g CO_2_ equivalent per kg tomatoes) in the research area vary largely from 34 in organic outdoor production, to 63 in organic polytunnels, to 99 in conventional polytunnels, and up to 1570 in heated greenhouses [5]. Additionally, outdoor production allows for more sustainable crop rotations. Despite the efficient use of resources, outdoor production in Central Europe was almost abandoned for commercial production after about 1990 because of more virulent genotypes of *Phytophthora infestans* (Mont.) de Bary, which cause late blight [6]. However, outdoor production has remained important for amateur gardeners. In Germany, amateur garden production accounts for about 50% of tomato production [7] but does not appear in the official statistics. In the UK, little or no commercial outdoor cultivation occurs [8], although outdoor tomatoes are widely grown by amateur gardeners [9]. The number of allotment plots in England alone exceeds 150000 [10].

In Germany, tomato cultivars are currently being developed by breeders who work according to organic standards [11], *e.g.*, Kultursaat e.V. (https://www.kultursaat.org), and by the Organic Outdoor Tomato Project [12, 13]. Organic breeding programs have increased their impact during the past 15 years in Europe [7]. Organic production is an alternative approach in agriculture and is aimed at producing food within a sustainable agroecosystem [14, 15]. However, at present, organic production relies to a large extent on crop cultivars from conventional high-input breeding backgrounds [16]. These cultivars may lack important traits required for stabilizing superior growth and yield when exposed to a suboptimal supply of nutrients and to increased stress by pests and pathogens in organic and/or low-input production [17–19] or when grown in home gardens. Consequently, the correct choice of appropriate cultivars as parents in breeding programs and for cultivation is a prerequisite for sustainable commercial and amateur cropping.

Tomato fruits appeal to our senses because of their rich array of flavours, shapes, sizes, and colours. Several quality parameters are particularly important for tomato production and consumer requirements. The tomato fruit should look appealing and be free of scratches, bruises, and signs of disease (outer quality). The internal quality of the tomato includes a balance with regard to its sugar content and acidity and the resulting acid-to-sugar ratio, its non-water-soluble fiber content and taste, its volatile organic substances, and its content of certain other substances such as the health-promoting lycopene [20]. The characteristics of sugar, acidity, and flavours may vary greatly with respect not only to the cultivar but also to the environmental conditions. The solar irradiation experienced during ripening, the harvesting time, and the temperature, water supply, and storage conditions are important parameters that influence internal quality parameters [20, 21]. Moreover, tomatoes are an integral part of the human diet being used fresh or cooked in pizza, ketchup, and tomato paste and provide important nutritional components such as carotenoids, vitamin C (ascorbic acid), phenols, and flavonoids. The carotenoid lycopene plays an especially important role for the consumer. Lycopene, which is highly abundant in tomatoes, is believed to provide health-promoting capabilities in the prevention and control of lung, breast, and prostate cancer [22]. Lycopene is also responsible for the red colour of tomatoes [23]. The mode of action of lycopene is that, together with vitamin C and vitamin E, it renders free radicals harmless [21]. Some studies have attempted to determine whether a link exists between the amount of biologically active substances and environmental conditions, such as drought stress, solar radiation, and suboptimal nutrient supply [24].

Breeding for yield and fruit quality may further increase the efficient use of resources. However, because of the limited possibilities for ambient control, a reduced heritability of important traits may be a major challenge to breeding programs. In-depth knowledge of quantitative genetic parameters and, in particular, genetic variance and heritability should help overcome those challenges.

The objectives of the study presented here were (i) to evaluate the variability of yield and fruit quality parameters in organic outdoor production, (ii) to calculate the heritability of these traits, (iii) to estimate the potential for successful selection based on the available genetic variability, and (iv) to identify superior genotypes for organic tomato production and breeding.

## 2. Material and Methods

### 2.1. Tomato Genotypes

24 indeterminate tomato genotypes were grown during 2015 and 2016 (Table 1) in a field trial at the research station for organic production of the University of Hohenheim, Kleinhohenheim, in southwest Germany. The first group of genotypes consisted of 16 cultivars of diverse origins. The second group consisted of eight breeding lines in the F_6_ generation of a single cross and both parent cultivars. The parents were also included in the first group. The cultivar Resi was selected as the parent because of its excellent fruit quality and moderate resistance against *P. infestans*, despite its being low-yielding. Phantasia F_1_ was selected as a parent because of its high yield and its high level of resistance against *P. infestans*, despite its poor fruit quality. During the preceding decade, breeding lines had been selected in generations F_2_ to F_5_ at multiple locations, including Kleinhohenheim, for yield, resistance against *P. infestans*, and fruit quality. The study presented here is part of the Organic Outdoor Tomato Project.

### 2.2. Yield, External Quality Parameter, and Internal (Biochemical) Quality

Yield was determined every week by harvesting mature healthy fruits. The external parameters were height, width, flesh firmness, and the number of fruit segments. The flesh firmness was measured with a Shore Durometer and scored four times across the tomato equator. Lycopene was measured in homogenate with 0.1% added antioxidant (butyl hydroxy toluene (BHT)) to prevent oxidation colour change using a Minolta CR-310 colorimeter (Minolta, Osaka). Prior to measurement, the Minolta CR-310 colorimeter was calibrated (*L*^∗^ = 91.1, *a*^∗^ = 0.39, and *b*^∗^ = 1.80). The lycopene concentration was calculated according to lycopene (*μ*g g^−1^ FW) = 5.8 + 3.735 × 10 − 5 a^∗^4.

Glucose and fructose concentrations were measured by the means of an enzymatic method with a kit from R-Biopharm AG (Germany). All samples were measured in triplicate. Organic acid concentration was determined in 10 mL of centrifuged (10 min at 12000 g) tomato homogenate; subsequently, a clear supernatant of the tomato homogenate was diluted with 30 mL of dH_2_O. The pH of the dilution was then measured by the use of an automatic titrator (TitroLine, Schott, Germany) in increments of added 0.1 M NaOH. The organic acid concentration was then calculated according to mg acid in aliquot = titrated volume of NaOH∗normality of NaOH (0.1)∗mEqwt (0.064 for citric acid)∗dilution factor. The soluble solid content (TSS in °Brix) was measured at 22°C using the Bausch and Lomb Abbe 3L refractometer (Bausch and Lomb Incorporated, Rochester, NY) in the clear homogenate.

### 2.3. Sowing, Planting, Location, and Climatic Conditions

Tomatoes were sown on April 6, 2015, and on April 8, 2016, in pots with a 10 cm diameter in the substrate Bio Potground (Klasmann KKS, Germany) in a greenhouse and were maintained until the plants reached a height of about 30 cm. Field trials were established on May 14, 2015, and on May 12, 2016, at the experimental station for organic agriculture at the University of Hohenheim, at Kleinhohenheim, according to certified organic standards [25]. This location is 435 m above sea level at 48°73′78^″^N and 9°20′06^″^E and has a sandy loam soil with high water holding capacity. In 2016, the preceding crop was clover grass; on May 10, 40 kg N ha^−1^ was applied as horn meal. The nitrogen situation in 2015 was similar, and the preceding crop was tomato. Plants were grown in three single rows from north to south on trellises and were each pruned to the main shoot. Complete replicates largely coincided with trellis rows. Weeds were removed by hoeing. The distance between rows was 4 m; the distance within the row was 0.5 m. The experimental layout in both years was a randomized complete block design with three replications.

In 2015 and 2016, precipitation during the growing season from May to October was 400 mm and 360 mm with a monthly mean of ca. 230 and 180 sunshine hours and a monthly average temperature of 16.2° and 15.7° C, respectively (Hohenheimer Klimadaten). The plants were irrigated as needed during the first 4 weeks after transfer to the field.

### 2.4. Linear Mixed Model

The data were assessed at the level of samples taken from individual tomato plants. If several samples were analysed per plant, we fitted the following linear mixed model:

(1) $$\begin{array}{c} y_{ijhkm}=\mu +g_{i}+r_{j}+t_{h}+p_{k}+q_{km}+e_{ijhkm}, \end{array}$$

where *y*_*ijhkm*_ is the response of the *i*-th genotype on the *m*-th plant on the *k*-th plot of the *j*-th replicate and *k*-th trellis row, *μ* is the overall mean, *g*_*i*_~*N*(0, *σ*_*g*_^2^) is the effect of the *i*-th genotype, *r*_*j*_ is the fixed effect of the *j*-th replicate, *t*_*h*_ is the fixed effect of the *h*-th trellis row, *p*_*k*_~*N*(0, *σ*_*p*_^2^) is the effect of the *k*-th plot, *q*_*km*_~*N*(0, *σ*_*q*_^2^) is the effect of the *m*-th plant on the *k*-th plot, and *e*_*ijhkm*_~*N*(0, *σ*_*e*_^2^) is the effect associated with the *ijhkm*-th sample. Our model has effects for both replicate and trellis rows because these two types of unit did not match perfectly and we did expect effects of trellis rows. A plot denotes a triplet of three plants for genotypes that were represented by three plants per replicate. For the other genotypes, a plot was identical with the single plant per replicate. In cases in which only a single sample was analysed per plant, we dropped the residual sample effect from the model (1). Variances of all random effects were estimated by residual maximum likelihood (REML). Occasionally, the estimate for a variance component converged to zero, indicating that the effect was not important. Phenotypic correlations were calculated on an entry-mean basis by using Plabstat Version 3Awin [26]. Assumptions of normality and homogeneity of variance were checked using diagnostic residual plots. When deviations were detected, a data transformation was considered. It turned out that a logarithmic transformation was needed in the cases of fruit weight and number of fruit segments.

### 2.5. Calculation of the Heritability

Heritability was estimated as

(2) $$\begin{array}{c} H^{2}=\frac{\sigma_{g}^{2}}{\sigma_{g}^{2}+0.5{\bar{v}}_{d}}, \end{array}$$

where ${\bar{v}}_{d}$ is the mean variance of a difference between adjusted genotype means [27]. This is a broad-sense heritability, reported to characterize the genetic variability in the tested material, under the replicated experimental design used. We emphasize that the reported estimates do not apply for early-generation selection of individual plants.

In order to obtain means for the three types of tomato (C = cocktail, S = salad, and F = beefsteak), a fixed main effect for type was introduced in model (1). Genotype means were tested for significant differences using the Tukey test with denominator degrees of freedom approximated using the Kenward-Roger method. Letter displays for mean comparisons were performed by using the method described in Piepho [28]. Due to the unbalanced data, the standard error of a difference is not constant across pairs and thus no common critical difference can be reported for the Tukey test.

For analysis across the two years, a random main effect for year was added; random effects for all effects present in (1), plus crosses with years, were also added. A crossed effect combining replicates and trellis rows was used to represent the two block factors for this joint analysis. Furthermore, because generally only one sample per plant was taken in the year 2016, whereas several samples were taken from some of the plants in the year 2016, we did not make a distinction between the sample and plant effects in the joint analysis, and hence, we dropped the plant effect from the model.

## 3. Results

For the sake of clarity, we concentrate on the mean values of both years.

### 3.1. Agronomical and Biochemical (Quality) Analysis

#### 3.1.1. Yield

**Figure 1:**
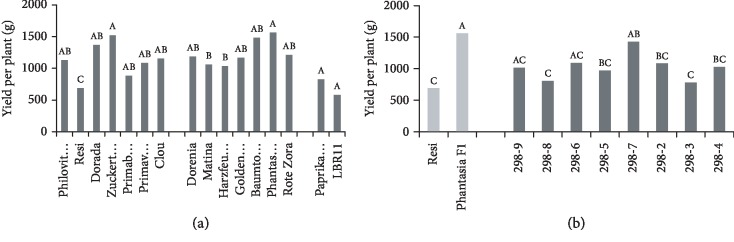
Yield. (a) Mean yield of 16 tomato cultivars during the years 2015 and 2016 in order of ascending fruit weight. Cocktail tomato (left block), salad tomato (centre), and beefsteak tomato (right). (b) Mean yield of F_6_ breeding lines of the cross Resi × Phantasia F_1_ and of both parents during 2015 and 2016 in order of ascending fruit weight. Means followed by a common letter are not significantly different at the 5% level in the Tukey test; in (a), the test was performed within each panel.

Significant differences in yield were observed (Figure 1(a)) at a moderate heritability of 0.56 (Table 2). Yield ranged from 576 g (LBR11) to 1560 g (Phantasia F_1_). No correlation was observed between fruit weight and yield (Table 3).

A large and significant difference in yield was observed between the parent cultivars (Figure 1(b)). All breeding lines grouped between the parents. The highest yielding breeding line 298-7 almost reached the yield of Phantasia F_1_. No correlation was observed between fruit weight and yield (Table 3).

### 3.2. Glucose and Fructose Concentrations

**Figure 2:**
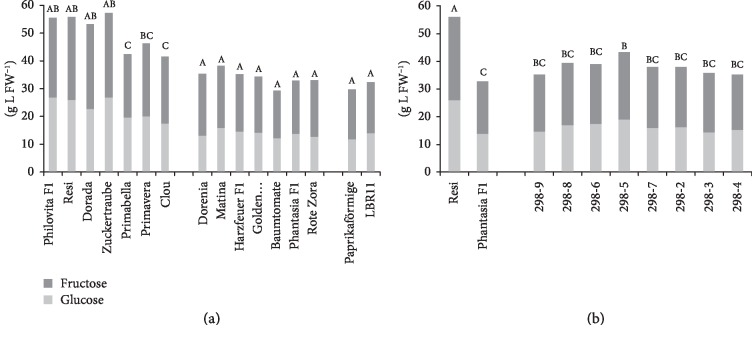
Glucose and fructose concentrations. (a) Mean glucose and fructose concentrations of 16 tomato cultivars during 2015 and 2016 in order of ascending fruit weight. Cocktail tomato (left block), salad tomato (centre), and beefsteak tomato (right). Mean glucose and fructose concentrations of F_6_ breeding lines of the cross Resi × Phantasia F_1_ and of both parents during 2015 and 2016 in order of ascending fruit weight. Means followed by a common letter are not significantly different at the 5% level in the Tukey test; in (a), the test was performed within each panel.

Significant differences in sugar concentrations were observed (Figure 2(a)), with a highly negative correlation of sugar contents with fruit weight (Table 3). Sugar concentration correlated with organic acid concentrations. Heritability was very high for glucose (0.92), fructose (0.81), and glucose+fructose (0.89) concentrations (Table 2). The heritability for °Brix (0.97) exceeded the heritability for the sugars. The Brix value is a measure of specific weights of mainly sugars, but also of other soluble substances. Within the salad tomato and within the beefsteak tomato cultivars, no significant differences in glucose and fructose contents were observed, respectively. The glucose concentration ranged from 26.9 g L FW^−1^ (Philovita F_1_) to 11.8 g L FW^−1^ (Paprikaförmige). The fructose concentration ranged from 26.9 g L FW^−1^ (Dorada) to 17.1 g L FW^−1^ (Paprikaförmige).

A large and significant difference in glucose and in fructose concentrations was observed between the parent cultivars (Figure 2(b)). All breeding lines grouped between the parents, but no breeding line reached the glucose and fructose concentrations of the parent Resi.

### 3.3. Lycopene Concentration

**Figure 3:**
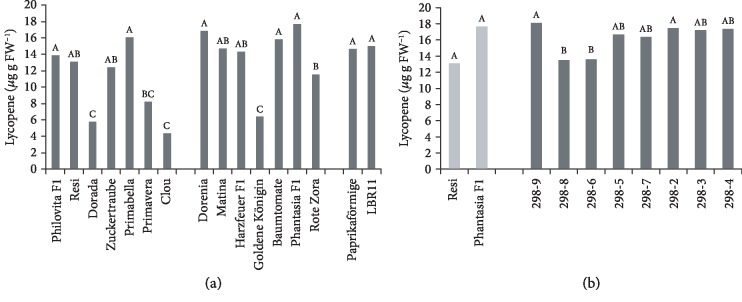
Lycopene concentration. (a) Mean lycopene concentration of 16 tomato cultivars during 2015 and 2016 in order of ascending fruit weight. Cocktail tomato (left block), salad tomato (centre), and beefsteak tomato (right). Mean lycopene concentration of F_6_ breeding lines of the cross Resi × Phantasia F_1_ and of both parents during 2015 and 2016 in order of ascending fruit weight. Means followed by a common letter are not significantly different at the 5% level in the Tukey test; in (a), the test was performed within each panel.

Significant differences in lycopene concentrations of the tomato genotypes were observed (Figure 3(a)), but no significant correlation of the lycopene concentration with fruit weight (Table 3) was detected. A weak negative correlation of lycopene with fructose concentration was noted. The heritability of lycopene was high (0.79, Table 2). As expected, the lycopene concentration was low in Dorada, Clou, and Goldene Königin, the cultivars with yellow fruits. The lycopene concentration varied fourfold and ranged from 17.7 *μ*g g FW^−1^ (Phantasia F_1_) to 4.4 *μ*g l FW^−1^ (Clou).

A difference in the lycopene concentrations of the two parents was observed (Figure 3(b)). All but one breeding line grouped between the parents, but many reached the lycopene concentration observed in Phantasia F_1_ and line 298-9 exceeded it slightly.

### 3.4. Organic Acid Concentration

**Figure 4:**
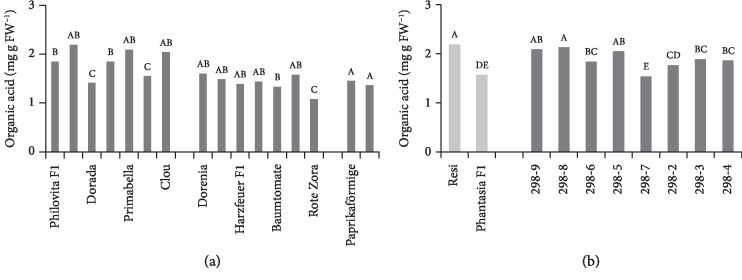
Organic acid concentration. (a) Mean organic acid concentration of 16 tomato cultivars during 2015 and 2016 in order of ascending fruit weight. Cocktail tomato (left block), salad tomato (centre), and beefsteak tomato (right). (b) Mean organic acid concentration of F_6_ breeding lines of the cross Resi × Phantasia F_1_ and of both parents during 2015 and 2016 in order of ascending fruit weight. Means followed by a common letter are not significantly different at the 5% level in the Tukey test; in (a), the test was performed within each panel.

Significant differences in organic acid concentrations were observed (Figure 4(a)) at very high heritability (0.93, Table 2), and the organic acid concentration correlated positively with sugar concentration and negatively with fruit weight and yield (Table 3). Organic acid concentrations ranged from 2.2 mg g FW^−1^ (Resi) to 1.1 mg g FW^−1^ (Rote Zora).

### 3.5. Sugar-to-Acid Ratio

**Figure 5:**
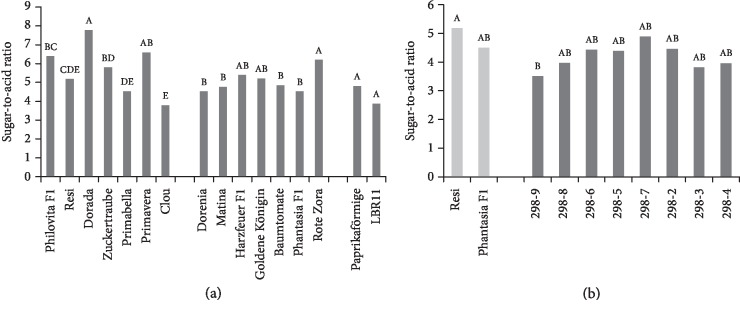
Sugar-to-acid ratio. (a) Mean sugar-to-organic acid ratio of 16 tomato cultivars during 2015 and 2016 in order of ascending fruit weight. Cocktail tomato (left block), salad tomato (centre), and beefsteak tomato (right). (b) Mean sugar-to-organic acid ratio of F_6_ breeding lines of the cross Resi × Phantasia F_1_ and of both parents during 2015 and 2016 in order of ascending fruit weight. Means followed by a common letter are not significantly different at the 5% level in the Tukey test; in (a), the test was performed within each panel.

Significant differences in the sugar-to-acid ratio were observed (Figure 5(a)). Heritability was high (0.78, Table 3). The organic acid concentration correlated negatively with the lycopene concentration (Table 3). Organic acid concentrations ranged from 7.78 (Dorada) to 3.79 (Clou).

The parent cultivars did not differ significantly in sugar-to-organic acid ratios (Figure 5(b)). All breeding lines other than 298-7 had lower sugar-to-organic acid ratios than the two parents.

## 4. Discussion

The heritability for traits related to taste, flavour, and health, *i.e.*, the concentrations of sugars, lycopene, and organic acids, was high to very high (Table 2). Therefore, for the screening and breeding of improved genotypes, a few environments may suffice to assess these traits. The same is true for traits related to outer quality, *i.e.*, fruit weight, fruit diameter, and number of fruit segments (Table 2). On the other hand, the medium heritability for yield suggests that trials in a larger number of environments are essential for reliable selection. It is acknowledged that our estimates of heritability are based on a single location and only assess genotype interactions with years. Hence, we could not estimate genotype-location interactions. It would be useful to conduct experiments at multiple locations in order to dissect genotype-location and genotype-year interaction effects.

In both years, infections by *P. infestans* were very few or absent. Thus, the conditions for quality assessment were ideal here, although the yield of individual genotypes may be influenced by late blight in other environments. A reduced yield would be expected, particularly for genotypes with low or no field resistance against *P. infestans* such as Zuckertraube, Matina, Harzfeuer F_1_, Goldene Königin, and Paprikaförmige.

The study of correlations between traits reveals challenges for the breeding for quality. Negative correlations of sugar concentrations with fruit weight and of organic acid concentrations with fruit weight and yield (Table 3) were frequently observed (e.g. [29, 30]) and suggest trade-offs in breeding for larger fruits and higher yields.

The concentrations of the primary metabolite sugars and organic acids were positively correlated. Breeding for an increased lycopene concentration, on the other hand, was not subject to these challenges (Table 3). The pigment lycopene is the main carotenoid in tomatoes and accumulates in high concentrations in mature red fruits. This compound has been associated with a reduced risk of chronic diseases such as cancer and heart diseases [22]. According to Arnao et al. [31], its antioxidative capacity is 1.2 times higher than that of *β*-carotene and 2.9 times higher than the antioxidant capacity of vitamin C (ascorbic acid). Our results indicate that intake can be increased by choosing a high lycopene cultivar. At present, new molecular tools are available for marker-assisted selection [32]. Surprisingly, no correlation was observed between fruit weight and yield (Table 3). This may be a result of the specific conditions in outdoor production with frequent changes in temperature and water supply levelling out the higher yield potential of genotypes with larger fruits.

Significant genetic variability was observed for all quality traits, thereby allowing growers to select cultivars based on consumer demands. However, variability was not always observed within each group of fruit type; *e.g.*, little variation for sugar concentration was observed in salad tomatoes and the sugar concentration was lower than that in cocktail tomatoes. Consumer preferences have evolved during the past decades, and cocktail tomatoes have gained increasing importance because of their visual appearance and quality attributes [33]. For instance, the share of small-fruited tomatoes increased from 21.7% in 2011 to 36.6% in 2017 in Germany [34]. Cocktail tomatoes are usually consumed raw, and hence, sensory attributes such as sweetness and juiciness and a fruitlike appearance have become more important. Breeding objectives for cocktail tomato cultivars have therefore been concentrated to a certain extent on quality aspects, possibly reducing yield potential. In contrast, salad tomato cultivars have been mainly bred for yield improvement, whereas quality traits have been considered less important [35, 36]. This has led to the higher sugar (Figure 2(a)) and organic acid (Figure 4(a)) concentrations of cocktail tomatoes observed in the present study. The lack of diversity within a group indicates some restrictions for present cultivation. For breeding programs, however, this is not a strong limitation, as crosses between groups can still be produced. Thus, the observed diversity can be used and the high heritability found in this study, representing the diversity across groups that were sampled in this study, suggests that there is scope for genetic improvement. In setting up new breeding efforts, it may be useful to estimate heritability for early-generation selection using data reflecting the experimental designs and genetic material to be used at that stage of variety development.

## Figures and Tables

**Table 1:** 16 tomato cultivars and 8 breeding lines tested in 2015 and 2016.

Genotype	Origin (cross, breeder)	Origin of seeds	Fruit colour	Fruit shape	Fruit weight (g)^1^	Fruit diameter (mm)^1^	No. of fruit segments^1^	Fruit type^2^
Philovita F_1_	De Ruiter	Volmary	Red	Round	12.9	27	1.9	Cocktail
Resi	OOTP^3^	Culinaris	Red	Round	13.7	30	2.0	Cocktail
Dorada	OOTP	Culinaris	Yellow	Round	16.6	32	2.0	Cocktail
Zuckertraube	Unknown	Bingenheimer Saatgut	Red	Round	18.4	33	2.0	Cocktail
Primabella	OOTP	Culinaris	Red	Round	18.9	32	2.4	Cocktail
Primavera	OOTP	Culinaris	Red	Round	19.7	33	2.0	Cocktail
Clou	OOTP	Culinaris	Yellow	Round	23.4	36	2.3	Cocktail
298-9	(Resi × Phantasia F_1_) F_6_	OOTP	Red	Round	32.5	37	2.0	Cocktail
298-8	(Resi × Phantasia F_1_) F_6_	OOTP	Red	Round	33.7	39	2.3	Cocktail
298-6	(Resi × Phantasia F_1_) F_6_	OOTP	Red	Round	43.2	45	3.0	Salad
298-5	(Resi × Phantasia F_1_) F_6_	OOTP	Red	Oblate	49.3	43	2.8	Salad
Dorenia	Kultursaat e.V.	Bingenheimer Saatgut	Red	Round	51.9	42	2.6	Salad
Matina	Hild	Culinaris	Red	Round	53.3	45	2.0	Salad
298-7	(Resi × Phantasia F_1_) F_6_	OOTP	Red	Round	54.6	45	3.4	Salad
298-2	(Resi × Phantasia F_1_) F_6_	OOTP	Red	Round	59.8	50	3.2	Salad
Harzfeuer F_1_	Institut für Züchtungsforschung Quedlinburg	Chrestensen	Red	Round	60.3	48	2.6	Salad
Goldene Königin	Unknown	Chrestensen	Yellow	Round	62.5	48	2.2	Salad
298-3	(Resi × Phantasia F_1_) F_6_	OOTP	Red	Round	64.1	47	2.9	Salad
Baumtomate	Zollinger	Zollinger	Red	Oval	65.7	43	2.1	Salad
298-4	(Resi × Phantasia F_1_) F_6_	OOTP	Red	Oblate	69.0	49	3.2	Salad
Phantasia F_1_	De Ruiter	Volmary	Red	Round	79.2	58	3.2	Salad
Rote Zora	Privates SamenArchiv	Culinaris	Red	Cylindrical	97.9	44	2.6	Salad
Paprikaförmige	Seed saver B. Rumkowski	Culinaris	Pink-red	Oblate	196.8	82	7.1	Beefsteak
LBR 11	Via seed savers; AVRDC	OOTP	Red	Oblate	201.8	76	6.5	Beefsteak

^1^Mean of 2015 and 2016. Heritability fruit weight (0.97), fruit diameter (0.97), and no. of fruit segments (0.86). ^2^Tomato type is defined by an average weight of <40 g for cocktail tomatoes, 40–100 g for salad tomatoes, and >100 g and >4 segments for beefsteak tomatoes. ^3^OOTP = Organic Outdoor Tomato Project.

**Table 2:** Estimates of variance components and heritability of traits related to fruit quality of 24 tomato genotypes under outdoor cultivation. Combined data of both years (2015 and 2016).

Trait	Variances^&^						*H* ^2^	Type means^$^		
	*σ*_*y*_^2^	*σ*_*y*×*g*_^2^	*σ*_*r*×*t*_^2^	*σ*_*p*_^2^	*σ*_*e*_^2^	*σ*_*g*_^2^				
Total yield (g plant^−1^)	82890	19776	2940	5115	122719	34641	0.556	1075.99^a^	1186.02^a^	951.65^a^
Brix (°)	0.0065	0.0137	0	0.0056	0.2508	1.369	0.970	7.8927^a^	6.1428^b^	5.4531^b^
Glucose (g L^−1^)	3.980	0.820	0.184	0	9.657	19.734	0.918	21.3367^a^	14.2545^b^	12.8357^b^
Fructose (g L^−1^)	2.386	2.456	2.113	0	15.197	14.778	0.811	26.3467^a^	20.0054^b^	18.3881^b^
Glucose and fructose (g L^−1^)	12.472	4.499	3.646	0	44.080	68.546	0.890	47.6840^a^	34.2757^b^	31.1878^b^
Total acid (mg L^−1^)	0.0789	0.00475	0.00126	0.00432	0.02759	0.08611	0.925	1.9021^a^	1.5588^b^	1.4315^b^
Sugar-to-acid ratio	0.0372	0.1907	0.0242	0.0137	0.9026	0.7551	0.780	5.3314^a^	4.5921^a^	4.2654 ^a^
Firmness (Shore)	17.168	4.184	0	0	40.601	23.907	0.761	35.3929^b^	41.8384^a^	39.2019^ab^
Fruit diameter (mm)	5.962	1.170	0	7.360	26.603	173.500	0.969	32.9914^c^	48.8618^b^	72.8228^a^
Fruit weight (g)^%^	0.0140	0.0314	—	—	—	0.6008	0.974	2.9880^c^	4.2153^b^	5.1965^a^
Number of fruit segments^%^	0.0082	0.02301	0.00087	0.00802	0.0227	0.1027	0.862	0.7402^c^	1.0599^b^	1.7750^a^
Lycopene (*μ*g g^−1^ FM)	16.329	1.255	0.099	1.751	15.051	10.207	0.788	11.7355^b^	15.2817^a^	15.5988^ab^

^%^Logarithmically transformed to stabilize variance between types. ^&^*y* = year, *y* × *g* = year by genotype, *r* × *t* = replicate within trial, *p* = plot, and *e* = residual error (plant).

**Table 3:** Spearman's rank correlation coefficients of traits related to fruit quality of 24 tomato genotypes under outdoor cultivation. Combined data for 2015 and 2016.

	Fruit weight	Yield	Glucose	Fructose	Glucose+fructose	Acid	Sugar-to-acid ratio
Yield	-0.041						
Glucose	-0.881^∗∗^	-0.043					
Fructose	-0.902^∗∗^	0.042	0.898^∗∗^				
Glucose and fructose	-0.897^∗∗^	-0.030	0.961^∗∗^	0.969^∗∗^			
Acid	-0.565^∗∗^	-0.428^∗^	0.534^∗∗^	0.469^∗^	0.561^∗∗^		
Sugar-to-acid ratio	-0.263	0.424^∗^	0.305	0.346	0.282	-0.501^∗^	
Lycopene	0.373	-0.175	-0.376	-0.429^∗^	-0.391	0.197	-0.528^∗∗^

^∗^ *p* = 0.05, ^∗∗^*p* = 0.01.

## Additional Points

*Key Message*. We report a high heritability of tomato quality traits such as organic acids, sugars, and lycopene and a medium heritability of yield in 24 genotypes with special emphasis on field cultivation.
